# Effects of acute caffeine ingestion combined with post-activation potentiation enhancement on the anaerobic capacity of male collegiate basketball players

**DOI:** 10.1080/15502783.2026.2670559

**Published:** 2026-05-12

**Authors:** Yongye Ma, Tianyuan He, Haoran Li, Qi Yan, Jian Sun, Duanying Li

**Affiliations:** a School of Athletic Training, Guangzhou Sport University, Guangzhou, Guangdong, China; b Key Laboratory of Human-Computer Intelligent Interaction for Athletic Performance and Health Promotion, Guangzhou Sport University, Guangzhou, Guangdong, China; c China Institute of Sport Science, Beijing, China; d Guangdong Provincial Key Laboratory of Human Sports Performance Science, Guangzhou Sport University, Guangzhou, Guangdong, China

**Keywords:** Caffeine, post activation potentiation enhancement, anaerobic capacity, basketball players

## Abstract

**Introduction:**

This study investigated the effects of an acute caffeine (CAF) supplementation strategy combined with post-activation potentiation enhancement (PAPE) on the anaerobic capacity of male collegiate basketball players.

**Methods:**

Using a randomized crossover design, 24 male collegiate basketball players (age: 20.29 ± 2.60 years) underwent three interventions: a standard warm-up (CON), PAPE with a placebo (PAPE+PLA), and PAPE with 5 mg/kg caffeine (PAPE+CAF). The PAPE protocol comprised a complex plyometric activation set including squat jumps, split jumps, and a 20-meter sprint. Anaerobic performance was assessed via the Wingate anaerobic test, measuring peak power (PP), average power (AP), total work (TW), fatigue index (FI), and the ratings of perceived exertion (RPE).

**Results:**

The results indicated that compared to CON, PAPE+PLA significantly enhanced PP, AP, and TW (*p* < 0.001). Furthermore, the PAPE+CAF intervention elicited further significant improvements in PP, AP, and TW compared to PAPE+PLA, indicating a clear synergistic effect (*p *<* *0.001). Although neither intervention significantly altered FI (*p* = 0.06), caffeine ingestion effectively reduced RPE (*p* < 0.001).

**Conclusion:**

This study confirms a synergistic effect between caffeine and PAPE, providing a theoretical foundation and practical reference for precompetition acute activation strategies for male basketball players.

## 1. Introduction

Maximizing athletic performance in high-intensity intermittent sports through acute interventions has emerged as a central topic in sports science in recent years [1]. Basketball is a court-based, confrontational sport characterized by high-intensity intermittent activity. The game rhythm consists of repeated bouts of maximal-intensity efforts alternating with brief rest periods, demanding superior explosive power and anaerobic endurance from players [2,3]. These attributes are core components of anaerobic capacity in basketball athletes and are critical factors influencing game outcomes [4–6]. Therefore, scientifically potentiating these capacities is a vital approach to enhancing on-court performance [7]. The advancements in sports science, particularly the integration of biomechanics and sports performance optimization research, have provided a theoretical and methodological foundation for developing more refined acute intervention strategies [8]. In this context, developing safe and effective acute potentiation strategies holds direct significance for competitive basketball performance.

Scientific warm-up strategies are crucial for enhancing athletic performance. Turki's study found that dynamic load warm-up can effectively improve athletes' ability to change direction repeatedly [9]. Additionally, the effectiveness of the postactivation potentiation effect induced by enhanced training has also been confirmed [10]. Its physiological mechanisms for enhancing anaerobic capacity involve myosin regulatory light chain phosphorylation, increased recruitment efficiency of high-threshold motor units, and modulation of tendon stiffness [11,12]. Sport-specific studies in basketball have shown that plyometric activation protocols incorporating squat jumps and split jumps can improve vertical jump height [13,14]. However, the PAPE effect exhibits significant inter-individual variability, and its optimal activation window is complexly modulated by training status, fast-twitch muscle fiber proportion, and accumulated fatigue [15].

Caffeine (1,3,7-trimethylxanthine) is classified as a Grade A evidence-level ergogenic aid by Sports Medicine Australia [16]. Multiple studies have confirmed that acute caffeine supplementation can significantly enhance various aspects of athletic performance. For example, moderate caffeine intake enhanced anaerobic abilities, such as vertical jump, agility, and reaction time, in female soccer players [17,18], and caffeine supplementation increased average power and lower limb functional strength in anaerobic sprint tests [19]. Furthermore, long-term combined supplementation of caffeine and exercise training (such as Zumba) has also been shown to synergistically improve functional performance in middle-aged women [20]. Meta-analyses confirm that acute caffeine supplementation can significantly enhance both explosive power and anaerobic endurance [1,21]. Its mechanisms of action mainly include antagonizing central adenosine A₁ receptors to reduce RPE and pain perception, increasing motor cortex excitability to promote the recruitment of high-threshold motor units, and stimulating ryanodine receptors to accelerate calcium ion release, thereby enhancing muscular strength and endurance [22–26]. However, excessive intake (>9 mg/kg) may cause tachycardia or gastrointestinal discomfort [27], and its efficacy is influenced by individual factors and circadian rhythms [28].

It is worth noting that PAPE and caffeine may amplify the activation effect through a synergistic interaction. PAPE primarily optimizes peripheral muscle fiber contractile efficiency, whereas caffeine enhances central neural drive. The combination of the two methods may produce a synergistic effect [29]. Research has found that soccer players ingesting caffeine combined with a PAPE protocol showed a significantly greater improvement in countermovement jump (CMJ) height compared to PAPE alone, with effects persisting for up to 5 min [29]. However, in female volleyball players, caffeine did not enhance the PAPE effect induced by back squats, possibly related to the upper-body dominant nature of the sport and gender-specific metabolic differences [30]. A recent study on boxers demonstrated that a 10-s cycling sprint PAPE protocol combined with caffeine resulted in significantly higher average power during a Wingate test compared to PAPE alone, alongside improved perceived exertion [31].

The high-intensity intermittent nature of basketball closely aligns with the physiological effects of both PAPE and caffeine. From an energy metabolism perspective, frequent jumping relies on rapid phosphocreatine replenishment, which PAPE may enhance by improving creatine kinase activity. The high-lactate environment during offense–defense transitions demands efficient glycolytic operation to clear metabolites, and caffeine has been shown to accelerate lactate transport [11]. Performance decline in the latter stages of a game is linked to central fatigue, which the adenosine antagonism of caffeine may help delay [32,33].

Nevertheless, a consensus on the synergistic effect of caffeine and PAPE has not been reached in the existing literature, likely due to sport-specific variations and a lack of combined interventions specifically designed for basketball [34]. Furthermore, traditional acute intervention studies often assess outcomes using a single movement (e.g. CMJ), failing to encompass the multifaceted anaerobic capacities required in basketball [2]. Conversely, the Wingate Anaerobic Test demonstrates a high degree of relevance for evaluating the core anaerobic metabolic capacities essential for basketball performance [35,36].

Therefore, this study employed a randomized crossover design comprising three interventions: a standard warm-up control (CON), PAPE combined with a placebo (PAPE+PLA), and PAPE combined with acute caffeine supplementation (PAPE+CAF). Using the Wingate anaerobic test, it aimed to compare the effects of these conditions on peak power, mean power, total work, fatigue index, and ratings of perceived exertion. The testing times were strictly controlled to eliminate potential circadian influences [28]. We hypothesized that: (1) the PAPE intervention would yield superior outcomes compared to the standard warm-up; and (2) caffeine ingestion would further enhance the benefits of PAPE, such that under the PAPE+CAF condition, participants' anaerobic performance would be significantly greater and their perceived exertion lower than under the PAPE+PLA condition.

## 2. Methods

### 2.1. Participants

This study focused on a male athlete cohort to circumvent the potential confounding heterogeneity in PAPE responses associated with sex [31], thereby enabling a more precise evaluation of the target interventions. The required sample size was estimated a priori using G*Power software (version 3.1.9.7, Germany). Based on an anticipated medium effect size (*p* = 0.27, derived from a previous meta-analysis comparing caffeine and placebo in Wingate tests [25]), an alpha level set at 0.05, a desired statistical power of 0.80, and an assumed correlation of 0.5 among repeated measures, the calculation determined a requirement of 24 participants.

Male collegiate basketball players from Guangzhou Sport University were recruited (see Table 1). According to the athlete classification framework proposed by McKay et al. [37], this cohort is classified as Tier 2. The inclusion criteria for participants were: (1) no history of neuromuscular or musculoskeletal disorders; (2) a minimum of 2 years of regular strength training experience; and (3) self-reported good health. Exclusion criteria comprised: (1) habitual smokers; (2) individuals reporting potential caffeine allergy or intolerance; and (3) those with an average daily caffeine intake exceeding 50 mg.

**Table 1.** Baseline characteristics of the participants.

	Mean ± SD
Age (years)	20.29 ± 2.60
Body mass (kg)	79.50 ± 10.52
Training experience (years)	3.96 ± 1.33
Average daily caffeine intake (mg)	35.26 ± 13.87

The study protocol was approved by the Ethics Committee of Guangzhou Sport University (Approval No.: 2025LCLL-119) and was registered as a clinical trial (Registration No.: ChiCTR2500108879). Each participant provided written informed consent after being fully informed of the study's purpose, procedures, and potential risks. They were also advised of their right to withdraw from the study at any time without penalty.

### 2.2. Experimental design

Prior to the formal testing phase, all participants attended a preliminary familiarization session. During this session, they received a detailed explanation of the experimental procedures, practiced all testing protocols, completed the informed consent process, and were screened using a Physical Activity Readiness Questionnaire (PAR-Q), with particular attention paid to any history of caffeine allergy or intolerance.

**Figure 1.**
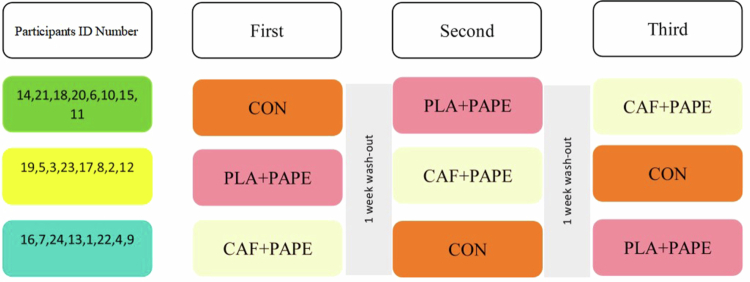
Flowchart of group allocation and experimental sequence (CON = control; PAPE+PLA = postactivation potentiation enhancement + placebo, PAPE+CAF = postactivation potentiation enhancement + caffeine).

To control for potential order effects, a Latin square design was employed to arrange the testing sequence. The 24 participants were randomly assigned to one of the balanced treatment sequences generated by this design using an online randomization program [38]. The fully randomized allocation scheme generated by this design randomly assigned the 24 participants to one of the three pre-set treatment sequences, with eight participants per sequence: sequence 1 (week 1: CON, week 2: PLA+PAPE, and week 3: CAF+PAPE), sequence 2 (week 1: PLA+PAPE, week 2: CAF+PAPE, and week 3: CON), and sequence 3 (week 1: CAF+PAPE, week 2: CON, and week 3: PLA+PAPE). This randomization schedule was generated and sealed by a researcher not involved in participant recruitment or testing, using a computer-based random number generator to ensure allocation concealment. Based on the half-life of caffeine (typically 2.5–4.5 h) and accounting for inter-individual variability in metabolism [1], a 1-week washout period was implemented between successive testing sessions to ensure sufficient elimination of caffeine effects (Figure 1).

**Figure 2.**
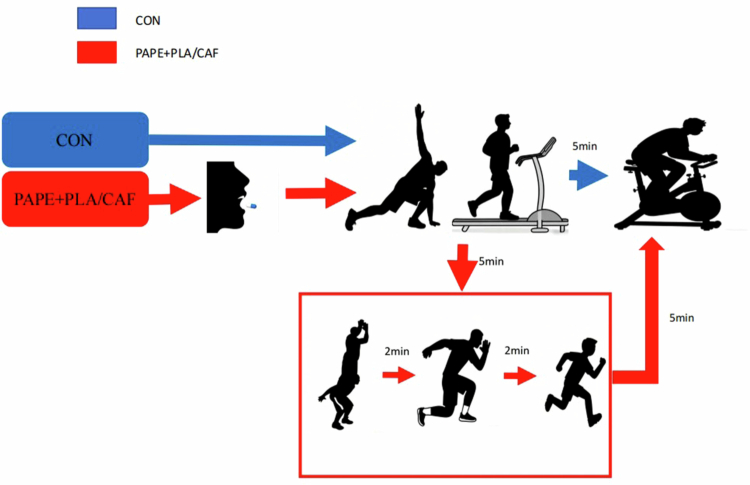
Flowchart of the intervention and testing procedures.

The CON group performed the 30-s Wingate maximal anaerobic power test immediately after a standard warm-up. During the caffeine and placebo testing sessions, participants ingested the respective capsules, dosed according to body mass, approximately 1 h before testing under double-blind conditions. The capsules contained 5 mg CAF/kg BM (Nutricost, USA) or an equivalent amount of maltodextrin filler as placebo (indistinguishable in appearance), aimed at achieving peak plasma caffeine concentrations during the testing period [1]. Approximately 30 min after ingesting the randomly assigned capsule, participants performed the test, participants performed a standardized warm-up, then executed the unified PAPE protocol (a complex plyometric set comprising squat jumps, split jumps, and a 20-m sprint). A 5-min rest period was observed postactivation [39], after which the 30-s Wingate Anaerobic Test was conducted (Figure 2).

**Figure 3.**
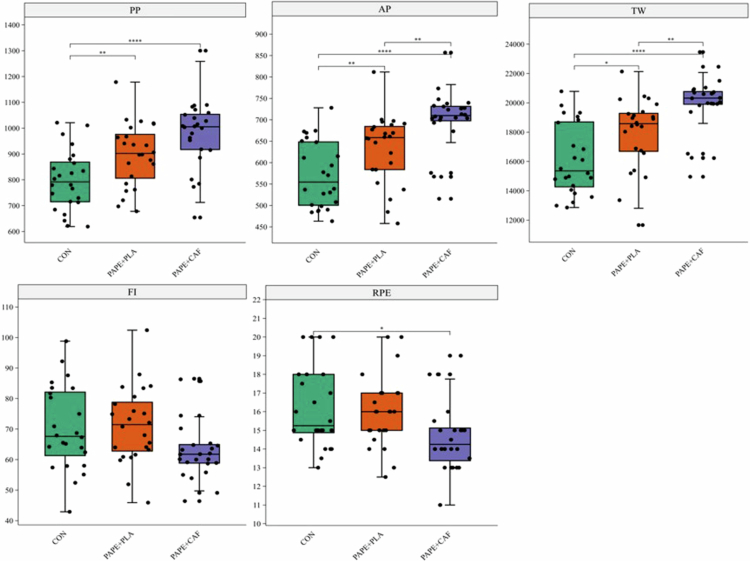
Results of the Wingate test and postexercise RPE under the three experimental conditions (The data are presented as box plots, where the box represents the interquartile range and the line inside the box indicates the median).

To maintain objectivity, the supplement allocation and administration process adhered strictly to double-blind principles. Both the experimental personnel conducting the tests and the participants were unaware of the capsule contents. All capsules were identical in appearance and weight. The randomization sequence was prepared and sealed by an independent staff member prior to the commencement of the study and remained concealed until the data analysis stage (Figures 1–3).

### 2.3. Warm-up and PAPE potentiation protocol

(1) **Standard Warm-up (CON session)**: Participants first completed 10 min of self-paced running (target distance ~1 km), followed by 5 min of dynamic stretching exercises targeting major lower-body muscle groups (e.g. walking lunges, high knees). (2) **Plyometric Potentiation Warm-up (used in PLA+PAPE and CAF+PAPE sessions)**: This protocol extended the standard warm-up (10 min running + 5 min dynamic stretching) by adding a specific plyometric activation complex. This complex comprised three exercises: squat jumps, split jumps, and a 20-m sprint. Each exercise was performed for two sets. The repetition scheme was eight repetitions per set for the squat jumps and split jumps, and one all-out sprint per set for the 20-m sprint. Rest intervals were strictly controlled: 60 s of rest was given between repetitions within a set, and 2 min of rest was provided between sets for the plyometric exercises.

All plyometric exercises were preceded by standardized instructional videos to ensure participants mastered the correct technique. The squat jump required participants to stand with feet shoulder-width apart, descend to a self-selected comfortable depth, and then perform an explosive vertical jump [40]. The split jump began from a staggered stance, requiring an explosive jump to switch leg positions in mid-air before landing [41]. The 20-m sprint required a maximal effort to cover the specified distance.

### 2.4. Anaerobic power test

Accurate power assessment requires that the testing method align with the movement pattern. Studies have pointed out that failure to meet the conditions of linear dynamics and complete force measurement may lead to significant errors [42]. The Wingate anaerobic test used in this study avoids this issue, as its power bicycle directly measures the torque and angular velocity output by the lower limbs, and power is calculated based on a well-established mechanical model (power = torque × angular velocity). This test has been widely validated as a reliable tool for assessing short-term maximal anaerobic capacity and fatigue rates [43], making it especially suitable for sports like basketball, which rely on high-intensity intermittent anaerobic energy systems. The test was conducted on a Monark 894E cycle ergometer (Monark Exercise AB, Sweden). Prior to testing, the resistance load on the ergometer was set at 7.5% of the participant's body weight (kg) [44]. The test duration was 30 s. Upon the tester's “go” command, the participant was required to reach maximal pedaling cadence as quickly as possible and maintain maximal effort throughout the entire test. Standardized verbal encouragement was provided by the researcher during the test to motivate participants to achieve their maximal potential. Immediately upon test completion, the participant's rating of perceived exertion (RPE) was recorded. The system automatically recorded power output data for each second. Based on the raw data, four primary variables were extracted and analyzed to comprehensively assess anaerobic capacity [35]: peak power (PP), average power (AP), total work (TW), and fatigue index (FI).

### 2.5. Pretest dietary standardization and experimental control

To ensure that all participants had highly consistent metabolic and physiological baselines across the three tests, allowing for precise evaluation of the independent and combined effects of the interventions, a strict pretest standardization process was implemented in this study. The protocol required participants to adhere to a uniform standardized diet template provided by the researchers for 24 h prior to each testing session, with meals consumed at the university cafeteria (Supplementary Material 1). The template specified the types of food, recommended portions, and meal timing for all three meals, based on a common diet for college students, with an emphasis on light cooking to ensure stable nutritional intake and minimize individual differences. Importantly, the protocol explicitly prohibited the consumption of any caffeine-containing foods (such as coffee, tea, energy drinks, and chocolate products) and alcohol within the 24 h preceding the test. Additionally, to ensure consistent testing conditions, participants were instructed to refrain from eating or drinking within 1 h before each test and to avoid any moderate- to high-intensity physical exercise within 48 h prior to testing. To strictly monitor compliance, each participant was required to complete a brief preparatory questionnaire before each test. Only data from participants who confirmed full adherence to all the above standardization protocols were included in the final analysis. To assess the effectiveness of blinding, after completing the PAPE+PLA or PAPE+CAF sessions, participants were asked to evaluate the substance they had ingested using the following question: “What do you think the capsule you consumed today contained?” The response options provided were: “caffeine,” “placebo,” and “I don’t know” (Supplementary Material 2).

### 2.6. Data analysis

Prior to the formal statistical analysis, we pre-established a data screening and handling process. For potential missing data, this study adopted the intention-to-treat principle, planning to include data from all randomly assigned participants who completed at least one test in the analysis. For potential outliers, we predefined that any data point exceeding the group mean ± 3 standard deviations would be considered a statistical outlier. If such outliers were detected, the original records would first be reviewed to ensure there were no data entry errors, and then robust statistical methods would be employed for sensitivity analysis to assess the impact of the outliers on the main conclusions. All quantitative data are presented as mean ± standard deviation (mean ± SD). If the distribution significantly deviated from normality, data were described using the median and interquartile range (IQR). The normality of residuals for each condition was assessed using the Shapiro–Wilk test. For data meeting the assumption of normality, a one-way repeated measures analysis of variance (factor: condition, 3 levels = CON, PAPE+PLA, and PAPE+CAF) was used. For data that do not follow a normal distribution, nonparametric tests were conducted. The Friedman test was used for overall comparison, and if significant, post-hoc pairwise comparisons were performed using the Wilcoxon signed–rank test. Sphericity was evaluated using Mauchly's test; if violated, the Greenhouse–Geisser correction was applied. In the event of a significant main effect, pairwise post-hoc comparisons with Bonferroni correction were performed to analyze differences between conditions. Effect sizes are reported as partial eta squared (*η*_p_²), with conventional thresholds for interpretation provided: small (0.01–0.06), medium (0.06–0.14), and large (≥0.14) [45]. The statistical significance threshold was set at *p* < 0.05. The success of blinding was assessed using the Bang Blinding Index [46]. All statistical analyses were performed using SPSS version 26.0 (IBM, Armonk, NY, USA).

## 3. Results

All 24 participants strictly adhered to the experimental protocol and completed all three tests, with no missing data. Outlier screening for the primary outcome measures was conducted based on the pre-established criterion (mean ± 3 standard deviations), and no extreme outliers requiring further handling were found in the data. Therefore, subsequent analyses were conducted using the complete dataset from all participants. During the caffeine intervention, participants reported no adverse events, such as palpitations or gastrointestinal discomfort, following the ingestion of the body mass-adjusted caffeine dose (5 mg/kg). In the PAPE+PLA condition, nine instances were correctly identified as placebo, while in the PAPE+CAF condition, ten instances were correctly identified as caffeine. The Bang's Blinding Index (BI) was used for quantitative assessment, yielding a BI of −0.25 for the PAPE+PLA group and −0.17 for the PAPE+CAF group. These results indicate that effective blinding was achieved regarding substance ingestion.

The results of the Shapiro–Wilk normality test for the primary outcome measures showed that PP, AP, TW, and FI data followed a normal distribution (*p* > 0.05), meeting the basic assumptions for parametric tests. Since the RPE data did not meet the normality assumption, the Friedman test was used. Post hoc comparisons were performed using the Wilcoxon signed–rank test.

The results of the Wingate test and postexercise RPE under the three experimental conditions are presented in Table 2 and Figure 3. Significant main effects of condition were found for peak power [F(2,46) = 63.96], average power [F(2,46) = 61.33], and total work [F(2,46) = 54.48; all *p* < 0.001]. No significant difference was observed for the fatigue index [*p* = 0.06; F(2,46) = 2.96]. The results of the post hoc pairwise comparisons are as follows: compared to CON, the PAPE+PLA intervention significantly improved PP (MD = 98.38 W, 95% CI [61.06, 135.71], *p* < 0.001), AP (MD = 61.48 W, 95% CI [34.19, 88.77], *p* < 0.001), and TW (MD = 1800.21 J, 95% CI [826.88, 2773.54], *p* < 0.001). PAPE+CAF showed a stronger enhancement effect. Compared to CON, PAPE+CAF showed the largest improvements in PP (MD = 179.10 W, 95% CI [129.80, 228.39], *p* < 0.001), AP (MD = 125.66 W, 95% CI [93.93, 157.39], *p* < 0.001), and TW (MD = 3752.13 J, 95% CI [2705.1, 4799.15], *p* < 0.001). More importantly, compared to PAPE+PLA, PAPE+CAF further significantly increased PP (MD = 80.71 W, 95% CI [45.96, 115.47], *p* < 0.001), AP (MD = 64.18 W, 95% CI [42.70, 85.66], *p* < 0.001), and TW (MD = 1951.92 J, 95% CI [1215.72, 2688.11], *p* < 0.001).

**Table 2.** Results of the 30-s Wingate Anaerobic Test and RPE.

Variable	CON	PAPE+PLA	PAPE+CAF	*p*	*η*_p_²
PP	798.87 ± 116.67	897.25 ± 123.05	977.96 ± 132.43	<0.001	0.74
AP	571.75 ± 77.72	633.23 ± 80.55	697.41 ± 73.75	<0.001	0.73
TW	16,132.79 ± 2424.08	17,933.00 ± 2434.40	19,884.92 ± 1992.03	<0.001	0.7
FI	70.31 ± 13.82	71.18 ± 12.48	63.63 ± 10.51	0.06	0.11
RPE	15.25 (14.75–18.00)	16.0 (15.00–17.00)	14.25 (13.25–15.25)	<0.001	–

CON = control, PAPE+PLA = postactivation potentiation enhancement + placebo, PAPE+CAF = postactivation potentiation enhancement + caffeine, PP = peak power, AP = average power, TW = total work, FI = fatigue index, RPE = rating of perceived exertion. Data are presented as mean ± SD for PP, AP, TW, and FI. RPE data, which were not normally distributed, are presented as median (IQR).

The results of the nonparametric tests showed that there were significant differences in RPE across the three conditions, *χ*²(2) = 19.74, *p* < 0.001, effect size Kendall's *W* = 0.41, indicating a medium-to-large treatment effect. Post hoc pairwise comparisons (Wilcoxon signed–rank test) with Bonferroni correction indicated that RPE under PAPE+CAF was significantly lower than under both the CON condition (MD = −1.75, 95% CI [−2.14, −0.86], *p* < 0.001) and PAPE+PLA condition (MD = −1.00, 95% CI [−1.79, −0.79], *p* < 0.001). However, there was no statistically significant difference between PAPE+PLA and CON conditions (MD = 0, 95% CI [−0.9, 0.48], *p* = 0.597).

## 4. Discussion

This randomized crossover study investigated the effects of acute caffeine supplementation combined with PAPE on the anaerobic capacity of male collegiate basketball players. The results indicated that, compared to the standard warm-up control condition, the plyometric PAPE protocol significantly enhanced PP, AP, and TW during the Wingate test. Furthermore, the co-ingestion of caffeine with the PAPE protocol led to a further significant improvement in PP, AP, and TW compared to PAPE alone. This result suggests that caffeine may have a synergistic effect with PAPE. However, neither intervention significantly altered the FI, whereas caffeine ingestion significantly reduced the rating of perceived exertion. These findings provide novel evidence for understanding the interactive mechanisms between neuromuscular potentiation and central regulation during anaerobic exercise and has supplemented the theoretical basis for pre-competition acute preparation strategies for male basketball players.

The significant enhancement in anaerobic power output metrics observed in the PAPE+PLA group compared to the control group can be explained by multiple physiological mechanisms underlying PAPE. PAPE, as a phenomenon in which prior high-intensity contractions enhance subsequent explosive performance, has been explained by several theoretical physiological mechanisms in the literature. Previous studies suggest that its mechanism may involve the phosphorylation of the myosin regulatory light chain (RLC), which may enhance the sensitivity of myofilaments to calcium ions and improve the contractile efficiency of muscle fibers [47]. Additionally, previous studies have demonstrated that plyometric activities can optimize motor unit recruitment patterns, particularly facilitating the mobilization of high-threshold, fast-twitch motor units, leading to greater power output within a short timeframe [48,49]. Similarly, the squat jumps, split jumps, and sprint exercises employed in this study are known to create more favorable mechanical conditions for subsequent anaerobic testing by enhancing neural drive and musculotendinous stiffness [49]. It is noteworthy that PAPE-induced neuromuscular adaptations are task-specific, and their effects are temporally dependent on the post-activation recovery window [39]. The decision to administer the Wingate test 5 min post-activation in this study aligns with the time point considered optimal for maximizing PAPE benefits while minimizing interference from fatigue [50]. Furthermore, recent studies suggest that plyometric training can further optimize energy transfer and power output in multi-joint movements by improving intramuscular coordination [51,52]. This training-induced improvement in neuromuscular adaptation and efficiency has also been demonstrated in sport-specific strength training studies. For example, a randomized controlled trial in handball players showed that 8 weeks of sport-specific explosive throwing training significantly increased the isokinetic peak torque of the shoulder rotator muscles and optimized functional strength ratios [53]. This study confirms the effectiveness of targeted training in improving neuromuscular coordination and power output. Therefore, the significant improvements in PP, AP, and TW observed in the PAPE group reflect not only peripheral mechanisms like RLC phosphorylation but also adaptive optimization at the level of neural movement control.

More importantly, the PAPE+CAF group demonstrated significantly superior PP, AP, and TW compared to the PAPE-only group, indicating a clear synergistic effect. To explain this synergistic effect, we referred to existing literature on the potential mechanisms underlying these two interventions. Caffeine has been reported to primarily act via central pathways, such as antagonizing adenosine receptors, to reduce perceived fatigue, enhance motivation, and increase neural drive [54–56], whereas the effects of PAPE are considered to be more peripheral, involving short-term optimization of muscle fiber contractile efficiency [22]. Therefore, the two interventions may exert complementary effects by acting at different physiological levels.

This complementarity may be particularly advantageous for tasks, such as the Wingate test, which relies heavily on the interaction between neural drive and muscular metabolic capacity [57,58]. It should be emphasized that the above mechanistic explanations are based on reasonable inferences from the literature and are intended to provide a theoretical perspective for the observed synergistic effects; the specific pathways require direct verification in future studies.

Furthermore, caffeine has been shown to accelerate calcium ion release from the sarcoplasmic reticulum and increase calcium transient amplitude by acting on ryanodine receptors [59]. This effect may have an additive or even synergistic interaction with the enhanced calcium sensitivity resulting from RLC phosphorylation during PAPE. Some studies also suggest that caffeine can improve muscular metabolic efficiency. For instance, by enhancing Na⁺/K⁺-ATPase activity and glycogenolysis rates, thereby delaying intracellular potassium accumulation and acidosis, which helps maintain cellular excitability and contractile function during very high-intensity exercise [24,59,60]. Consequently, the combined application of caffeine and PAPE not only acts concurrently on both central and peripheral levels but may also amplify the potentiating effect through multi-target interactions at the molecular and cellular levels, ultimately manifesting as the observed further improvement in PP, AP, and TW.

However, neither PAPE alone nor in combination with caffeine significantly improved the fatigue index. FI reflects the rate of decline in anaerobic power output, primarily rooted in the depletion of phosphagens, intracellular acidosis due to hydrogen ion accumulation, and inhibition of neuromuscular transmission [61]. Although PAPE can optimize the initial phase of power output, its effect may be insufficient to counteract the extreme metabolic acidosis and ionic homeostasis disruption occurring in the latter half of the Wingate test [62]. Similarly, while caffeine has been reported to have certain metabolic modulatory effects (e.g. promoting lactate clearance and fatty acid oxidation), its role during maximal anaerobic exercise may lie more in increasing the absolute power output rather than altering the rate of fatigue development [63,64]. On the other hand, caffeine ingestion significantly reduced participants' RPE, a result consistent with previous research [65]. This finding aligns with a recent study in tennis players, where caffeine supplementation similarly led to significantly lower RPE scores during high-intensity training [66]. As a central nervous system stimulant, caffeine attenuates the activation of fatigue signaling pathways by blocking adenosine receptors, thereby altering the brain's interpretation of effort perception and physiological strain [2,67]. This suggests that even at comparable levels of physiological fatigue, caffeine can help athletes better tolerate the discomfort associated with high-intensity exercise by modulating the perceptual level, which holds particular significance for sports like basketball that require repeated sprints and rapid decision-making [68]. Furthermore, the ergogenic effect of caffeine on short-duration, high-intensity performance is corroborated by recent evidence from other athletic domains, such as its efficacy in improving sprint time in swimmers [69], which aligns with the power output enhancement observed in the present Wingate test.

In summary, the findings of this study demonstrate a synergistic effect of acute caffeine supplementation combined with a plyometric PAPE protocol on enhancing the anaerobic capacity of male collegiate basketball players, with pronounced benefits for improving PP, AP, and TW. The underlying mechanism can be attributed to the effective complementarity and superposition of the central effects of caffeine and the peripheral mechanisms of PAPE. Although the combination did not alter the fatigue index, caffeine's role in reducing perceived exertion remains of practical value. Future research could further explore the application of this combined strategy across different sports, genders, and training levels, as well as the adaptive responses to its long-term use.

### 4.1. Limitations

This study has a blinding limitation in the CON condition due to the absence of capsule ingestion and differences in procedures. However, the main conclusions are based on the strictly double-blind comparison between PAPE+CAF and PAPE+PLA (validated by the Bang Blinding Index), and therefore, this limitation does not affect the primary inference that a synergistic effect exists between caffeine and PAPE. It should be acknowledged that while this study controlled for daily caffeine intake (<50 mg/day) to mitigate tolerance interference, genotyping was not performed. Consequently, some individuals with slower caffeine metabolism may not have reached optimal plasma concentrations, potentially limiting the strength of the observed synergistic effect. Moreover, future research should incorporate direct measurement approaches, such as neurophysiological assessments and muscle biopsies, to elucidate the potential central and peripheral complementary mechanisms underlying these effects. Additionally, the outcome measures were centered on anaerobic capacity indices; derived parameters, such as post-exercise recovery rates, were not assessed.

## 5. Conclusion

This randomized crossover study systematically investigated the effects of acute caffeine supplementation combined with a plyometric postactivation potentiation enhancement protocol on the anaerobic capacity of a limited sample of​ male collegiate basketball players. The results suggest that, compared to a standard warm-up, both the PAPE+CAF and PAPE+PLA interventions exhibited a more potent effect in enhancing PP, AP, and TW during the Wingate test. Notably, the PAPE+CAF intervention produced greater improvements in these measures than PAPE alone,​ indicating a synergistic effect between caffeine and PAPE on anaerobic performance. Although neither intervention significantly altered the FI, caffeine effectively enhanced exercise tolerance by reducing ratings of perceived exertion. This study provides a practical acute pre-competition preparation strategy for male basketball players. However, these observations warrant confirmation in future studies with larger and more diverse cohorts.

## Supplementary Material

Supplementary_Material_2 Supplementary_Material_2.

Supplementary_Material_1 Supplementary_Material_1
