# Supplementary material for: Effects of acute caffeine ingestion combined with post-activation potentiation enhancement on the anaerobic capacity of male collegiate basketball players
Source: J Int Soc Sports Nutr. 2026 May 12;23(1):2670559. doi: 10.1080/15502783.2026.2670559 (PMC13169452; doi:10.1080/15502783.2026.2670559)
Supplement: Supplementary_Material_2 [file RSSN_A_2670559_SM1618.docx]

**Blinding Design and Validity Verification**

(This material details the methods, tools, data recording, and analysis results employed in this study to ensure the effectiveness of the blinding design.)

**Blinding Verification Questionnaire and Implementation Procedure**

To immediately and independently assess participants' guesses regarding the intervention measures after each test, a standardized questionnaire was designed. The questionnaire was completed independently by participants in an undisturbed setting within 5 minutes after the full procedure of the PAPE+PLA and PAPE+CAF tests.

**Questionnaire Format:​**

**Post-Exercise Sensation and Capsule Content Blinding Verification Questionnaire**

Dear Participant,

Hello!

Based on your overall physical sensations during the test you have just completed, please make your best guess regarding the content of the capsule taken before the test. There are no right or wrong answers—your response is solely for scientific research, completely anonymous, and will not affect you in any way. Please select the one and only​ option below according to your genuine feelings.

Participant ID:​ ______

Test Date:

Question:Based on your physical sensations before this test, what do you think was most likely inside the capsule taken prior to the test?

[ ] A. Caffeine​

[ ] B. Placebo (substance without caffeine)​
[ ] C. I don’t know

End of questionnaire. Thank you for your cooperation!

**Data Recording**

| Participant ID | Actual Intervention Condition | Participant Guess | Guess Correct? |
| --- | --- | --- | --- |
| 1 | PAPE+CAF | CAF | Yes |
| 1 | PAPE+PLA | PLA | Yes |
| 2 | PAPE+PLA | CAF | No |
| 2 | PAPE+CAF | PLA | No |
| 3 | PAPE+PLA | CAF | No |
| 3 | PAPE+CAF | CAF | Yes |
| 4 | PAPE+CAF | CAF | Yes |
| 4 | PAPE+PLA | PLA | Yes |
| 5 | PAPE+PLA | CAF | No |
| 5 | PAPE+CAF | PLA | No |
| 6 | PAPE+PLA | CAF | No |
| 6 | PAPE+CAF | CAF | Yes |
| 7 | PAPE+CAF | PLA | No |
| 7 | PAPE+PLA | PLA | Yes |
| 8 | PAPE+PLA | CAF | No |
| 8 | PAPE+CAF | PLA | No |
| 9 | PAPE+CAF | CAF | Yes |
| 9 | PAPE+PLA | CAF | No |
| 10 | PAPE+PLA | PLA | Yes |
| 10 | PAPE+CAF | PLA | No |
| 11 | PAPE+PLA | CAF | No |
| 11 | PAPE+CAF | CAF | Yes |
| 12 | PAPE+PLA | CAF | No |
| 12 | PAPE+CAF | PLA | No |
| 13 | PAPE+CAF | CAF | Yes |
| 13 | PAPE+PLA | CAF | No |
| 14 | PAPE+PLA | CAF | No |
| 14 | PAPE+CAF | PLA | No |
| 15 | PAPE+PLA | PLA | Yes |
| 15 | PAPE+CAF | PLA | No |
| 16 | PAPE+CAF | CAF | Yes |
| 16 | PAPE+PLA | CAF | No |
| 17 | PAPE+PLA | CAF | No |
| 17 | PAPE+CAF | PLA | No |
| 18 | PAPE+PLA | PLA | Yes |
| 18 | PAPE+CAF | PLA | No |
| 19 | PAPE+PLA | CAF | No |
| 19 | PAPE+CAF | CAF | Yes |
| 20 | PAPE+PLA | CAF | No |
| 20 | PAPE+CAF | PLA | No |
| 21 | PAPE+PLA | PLA | Yes |
| 21 | PAPE+CAF | PLA | No |
| 22 | PAPE+CAF | PLA | No |
| 22 | PAPE+PLA | PLA | Yes |
| 23 | PAPE+PLA | CAF | No |
| 23 | PAPE+CAF | CAF | Yes |
| 24 | PAPE+CAF | PLA | No |
| 24 | PAPE+PLA | PLA | Yes |

**Blinding Validity Analysis Contingency Table**
Note: This table summarizes the guessing results of all 24 participants in the two interventions, PAPE+PLA and PAPE+CAF.

| Actual Intervention | Guessed CAF | Guessed PLA | Total |
| --- | --- | --- | --- |
| PAPE+CAF | 10 | 14 | 24 |
| PAPE+PLA | 15 | 9 | 24 |
| Total | 25 | 23 | 48 |
