# Supplementary material for: Effects of acute caffeine ingestion combined with post-activation potentiation enhancement on the anaerobic capacity of male collegiate basketball players
Source: J Int Soc Sports Nutr. 2026 May 12;23(1):2670559. doi: 10.1080/15502783.2026.2670559 (PMC13169452; doi:10.1080/15502783.2026.2670559)
Supplement: Supplementary_Material_1 [file RSSN_A_2670559_SM1607.docx]

**24-Hour Standardized Preparation Guidelines Before Anaerobic Capacity Testing**

Dear Participant,

To ensure that the data from each test are accurate and comparable, please strictly follow the guidelines below for your diet and activities 24 hours before each test. The goal is to keep your body in a very similar state before all three tests.

**Prohibited Items (Critical):**

Any caffeine-containing products: such as coffee, tea, all tea beverages, cola, energy drinks, chocolate, and related snacks.Any alcoholic beverages.

**Standardized Dietary Arrangement:**

Dinner the Night Before the Test: Choose a light meal at the campus cafeteria. Recommended combination:Rice (about 150-200 grams, equivalent to about 2 fist-sized portions),One source of high-quality protein (e.g., chicken breast, steamed fish, lean beef, or tofu, about 100-150 grams, equivalent to the size of one palm),Two types of vegetables (e.g., broccoli, spinach, lettuce, etc., stir-fried or blanched, totaling about 200 grams, equivalent to about 2 fist-sized portions)

Avoid fried, braised, spicy, or overly salty dishes.

**Breakfast on the Test Day:**

Must be completed 2 hours before the test. Recommended combination:

Carbohydrate source (2 slices of whole wheat bread or 1 steamed bun or 50 grams of instant oatmeal mixed with hot water),Protein source (1 boiled egg + 250 milliliters of plain milk or unsweetened soy milk),Fruit (1 banana or 1 apple).

**Snacks and Hydration on the Test Day:**

If you feel hungry in the morning, you may have 1 banana or 1 slice of whole wheat bread. Only drink plain water throughout the day. Please note: Do not eat within 1 hour before the test, and avoid drinking large amounts of water (small sips are allowed to moisten your throat).

**Activity and Sleep Requirements:**

Avoid intense activities such as soccer, basketball, high-intensity workouts, and long-distance running within 48 hours before each test.Maintain a regular sleep schedule, ensuring 7-8 hours of quality sleep the night before the test.

**Compliance Confirmation:**

After checking in for each test, you will be required to fill out a brief Pre-test Preparation Questionnaire to confirm your adherence to all the above guidelines. Your careful cooperation is the foundation of the success of this study, and we sincerely thank you!
